# Will non-invasive methods replace bone biopsy in patients with chronic kidney disease?

**DOI:** 10.1590/2175-8239-JBN-2025-0385en

**Published:** 2026-03-30

**Authors:** Daniel Souza Gonçalves de Araujo, Claudio Oka Lobo, Francisco Ribeiro Barbosa

**Affiliations:** 1 Universidade de São Paulo, Faculdade de Medicina, São Paulo, SP, Brazil.

Dear Editor,

I read with great interest the study conducted by Diz-Lopes et al.^1^, recently published in this journal, which investigated the utility of FRAX^®^ and bone biomarkers for predicting fractures in patients with chronic kidney disease (CKD) in the pre-dialysis phase. I highlight the relevant initiative to validate accessible tools like FRAX^®^ in this specific population, demonstrating its diagnostic accuracy even in the absence of bone mineral density (BMD) data.

However, the absence of a significant association between histomorphometric subtypes of renal osteodystrophy and the incidence of fractures, a central finding of the study, warrants further reflection when the discussion is applied to the Brazilian context. Although the data suggest a secondary role for biopsy in the prediction of fractures in the pre-dialysis setting, caution is necessary to avoid decreeing, by generalization, the obsolescence of bone biopsy.

The relevance of biopsy in Brazil transcends fracture prediction; it resides in its unique ability to diagnose metal intoxication. As evidenced by Coutinho et al.^2^, the prevalence of aluminum toxicity in hemodialysis cohorts in the Brazilian Northeast remains alarming, reaching 46.8% of the analyzed samples. This scenario contrasts drastically with the low incidence of aluminum bone disease reported in North American and European registries, the context in which the case series of Diz-Lopes is situated. Recently, Oliveira et al.^3^ analyzed this discrepancy and the possible causes for the persistence of this phenomenon in our setting.

The risk of neglecting the invasive method lies in its therapeutic implications. A patient identified as “high risk” by FRAX^®^ would be a candidate for antiresorptive therapy. However, should this patient present aluminum deposits—a condition that would not be detected by FRAX^®^, BMD, or turnover biomarkers—the introduction of potent inhibitors of bone resorption could precipitate or aggravate severe adynamic bone disease, preventing the mobilization of the metal from the mineral compartment and, paradoxically, increasing bone fragility.

Therefore, the dissociation between histomorphometry and fractures observed by Diz-Lopes should not be interpreted as a failure of the invasive method, but perhaps as a reflection of a population in which mineral toxicity is rare. In the Brazilian context, bone biopsy offers the crucial distinction between high-turnover osteoporosis and osteomalacia or toxic adynamic bone disease, which require a distinct therapeutic approach, such as chelation with desferrioxamine.

In conclusion, we advocate that bone biopsy retains its clinical relevance beyond its role in research, even in the context of the incorporation of FRAX^®^ and new biomarkers into the nephrologist’s arsenal for patient screening. The current evidence used to justify replacing this technique may be based on studies that do not necessarily reflect the Brazilian context and, therefore, ignore possibilities such as the underdiagnosis of aluminum deposition.
