# Supplementary material for: Will non-invasive methods replace bone biopsy in patients with chronic kidney disease?
Source: J Bras Nefrol. 2026 Mar 30;48(3):e20250385. doi: 10.1590/2175-8239-JBN-2025-0385en (PMC13034618; doi:10.1590/2175-8239-JBN-2025-0385en)
Supplement: Translation

# Translation: Os métodos não invasivos substituirão a biópsia óssea em pacientes com doença renal crônica?

**DOI:** 10.1590/2175-8239-JBN-2025-0385pt

**Authors:** Daniel Souza Gonçalves de Araujo, Claudio Oka Lobo, Francisco Ribeiro Barbosa

**Affiliations:** 1 Universidade de São Paulo, Faculdade de Medicina, São Paulo, SP, Brasil.

Caro Editor,

Li com grande interesse o estudo conduzido por Diz-Lopes et al.^1^, recentemente publicado neste periódico, que investigou a utilidade do FRAX^®^ e de biomarcadores ósseos na predição de fraturas em pacientes com doença renal crônica (DRC) na fase pré-diálise. Destaco a relevante iniciativa de validar ferramentas acessíveis como o FRAX^®^ nessa população específica, demonstrando sua acurácia diagnóstica mesmo na ausência de dados de densidade mineral óssea (DMO).

Entretanto, a ausência de associação significativa entre os subtipos histomor­fométricos de osteodistrofia renal e a incidência de fraturas, achado central do estudo, merece reflexão quando transpomos a discussão para a realidade brasileira. Embora os dados sugiram um papel secundário para a biópsia na predição de fraturas no cenário pré-dialítico, é importante ter cautela para não decretar, por generalização, a obsolescência da biópsia óssea.

A relevância da biópsia no Brasil transcende a predição de fraturas; ela reside em sua competência única para o diagnóstico de intoxicação por metais. Conforme evidenciado por Coutinho et al.^2^, a prevalência de toxicidade por alumínio em coortes de hemodiálise no Nordeste brasileiro permanece alarmante, atingindo 46,8% das amostras analisadas. Esse cenário contrasta drasticamente com a baixa incidência de doença óssea por alumínio reportada em registros norte-americanos e europeus, contexto no qual se insere a casuística de Diz-Lopes. Recentemente, Oliveira et al.^3^ analisaram tal discrepância e as possíveis causas para a persistência desse fenômeno em nosso meio.

O risco de negligenciar o método invasivo reside nas implicações terapêuticas. Um paciente identificado como de “alto risco” pelo FRAX^®^ seria candidato à terapia antirreabsortiva. No entanto, caso esse paciente apresente depósitos de alumínio, condição que não seria detectada pelo FRAX^®^, pela DMO ou pelos biomarcadores de *turnover*, a introdução de potentes inibidores da reabsorção óssea poderia precipitar ou agravar uma doença óssea adinâmica severa, impedindo a mobilização do metal do compartimento mineral e aumentando, paradoxalmente, a fragilidade óssea.

Portanto, a dissociação entre histomorfometria e fraturas observada por Diz-Lopes não deve ser interpretada como uma falha do método invasivo, mas talvez como reflexo de uma população na qual a toxicidade mineral é rara. No cenário brasileiro, a biópsia óssea oferece a distinção crucial entre osteoporose de alto *turnover* e osteomalácia ou doença adinâmica tóxica, que exigem abordagem distinta, como a quelação com desferroxamina.

Em conclusão, defendemos que a biópsia óssea conserva sua relevância clínica além de seu papel em pesquisa, mesmo no contexto da incorporação do FRAX^®^ e de novos biomarcadores no arsenal do nefrologista para a triagem de pacientes. A atual evidência existente para suplantar o uso dessa técnica pode estar baseada em estudos que não refletem necessariamente a realidade brasileira e, portanto, ignoram possibilidades como o subdiagnóstico de deposição de alumínio.
